# Community Environment Co-Production and Environmental Satisfaction of Older Urban Residents in Shanghai, China

**DOI:** 10.3390/ijerph20032684

**Published:** 2023-02-02

**Authors:** Feng Jiang, Jing Wang, Lufa Zhang, Jin Luo, Li Li, Ruilong Wu

**Affiliations:** 1 School of International and Public Affairs, Shanghai Jiao Tong University, 1954 Huashan Road, Xuhui District, Shanghai 200030, China; 2 Institute of Healthy Yangtze River Delta, Shanghai Jiao Tong University, 1954 Huashan Road, Xuhui District, Shanghai 200030, China; 3 China Institute for Urban Governance, Shanghai Jiao Tong University, 1954 Huashan Road, Xuhui District, Shanghai 200030, China; 4 Shanghai Municipal Center for Senior Citizens Programs Development, 339 Luding Road, Putuo District, Shanghai 200062, China

**Keywords:** co-production, environmental satisfaction, associated factors, urban resident, China

## Abstract

Objective: Many factors may affect the environmental satisfaction of elderly people, including their sense of involvement. This study examined the associations between community environment co-production and environmental satisfaction in older urban residents in China. Methods: A cross-sectional survey was conducted in four age-friendly communities in Shanghai, China. Co-production and environmental satisfaction were assessed through a self-developed questionnaire. General health status was measured through the EuroQol-Visual Analogue Scale (EQ-VAS). Data on affective commitment for the community demographic and health-related factors were also collected. Multilevel linear regression was used to detect the associations. Results: In total, 480 older urban residents completed the survey. On average, the environment satisfaction score was 76.82/90, 8/10 for co-production, and 87.5/100 for EQ-VAS. Univariate analysis demonstrated environmental satisfaction was associated with educational background, party membership, physical activity, community location, age, sleep hours, co-production, affective commitment, and EQ-VAS. After controlling for confounding factors, the co-production score was significantly associated with higher environmental satisfaction (β = 4.68, *p* < 0.001). Multiple linear regression revealed that effective commitment for the community (β = 6.17, *p* < 0.001) and EQ-VAS (β = 0.06, *p* = 0.002) were also significantly associated with environment satisfaction. Conclusion: Community environment co-production was positively associated with environmental satisfaction among older urban residents in Shanghai. Environmental co-production should be encouraged when developing age-friendly communities for the elderly.

## 1. Introduction

Population aging is becoming a major challenge for countries all around the world. According to the United Nations, the population aged 60 or above will reach 2.5 billion, or 21.3% of the total population, by 2050 [1], with 43.2% of the elderly population living in cities [2]. 

China has the largest aging population in the world. According to the Seventh National Population Census, by the end of 2020, 264 million Chinese aged 60 or above, accounting for 18.7% of the total population [3]. At the same time, there were 63.89% of Chinese people lived in cities [4]. Aging in urban cities is a crucial challenge for governance, especially in large cities like Shanghai. According to the Shanghai Statistical Yearbook 2021, there were 5.81 million (23.38%) citizens aged 60 or above in Shanghai and 16.3% aged 65 or above [5].

To promote the health and well-being of the elderly population, the World Health Organization (WHO) developed the ‘age-friendly’ concept, which contains three domains, physical environment (e.g., transportation, housing, outdoor spaces, and buildings), service provision (e.g., social and health services, communication and information), and social environment (e.g., civic participation, respect, and social inclusion) [6]. This conceptual framework has become one of the most popular tools for assessing age-friendly cities and communities. To encourage the dissemination of its work, the WHO launched the Age-friendly Cities and Communities Program and called for political action to create age-friendly environments for the elderly [7,8,9]. For the project to be successful, elderly people must be involved as partners. For example, they can play an important role in suggesting, implementing, and monitoring improvements in the community’s environment. Such interaction can be enhanced through co-production, which leads to mutual reinforcement and transformation of societal outcomes [10].

Co-production has been widely recognized since its introduction in the 1970s [11]. In general, any involvement of citizens in public governance or delivery can be defined as co-production [12]. In public service, co-production can occur in stages of production, including design, planning, delivering, managing, monitoring, and evaluation [13,14,15]. The co-production process aims to maximize the efficiency of resources and contributions of different stakeholders, individually or collectively [16,17]. There are more participants in the co-production of social policy processes, especially end-users [17]. Any individual can contribute to co-production independently with the government’s support or collaborate with the government [18]. Collaborations in the co-production process can be with other individuals or organized groups. As co-production may improve the quality and quantity of public services, including environment governance [19], it has become a focus of public administration studies. 

Desired outcomes of co-production range from the improved process to new outputs and finally to long-term consequences, e.g., to meet the needs of users [16,20]. The ultimate outcomes of co-production can impact the individuals, communities, and even the whole system, while the actual results of co-production vary widely across the literature. In environmental governance practices, co-production can increase the adaptive capacity in Arctic communities [21,22], improve community safety [23] and solid waste management [24], and enhance environmental satisfaction.

Environmental satisfaction refers to the individual appraisal of their residential environment based on their needs, expectations, and achievements [25]. It is a complex construct since it depends on the place, culture, and resident evaluations [26]. Previous studies showed that satisfaction was constructed by the dimensions of satisfaction for urban policy and planning, design principles, and social milieu [27,28,29,30,31,32]. There are three main perspectives regarding satisfaction with the community environment [33]: the degree to which the environment facilitates user goals [25], the gap between individual actual and aspired needs [34], and the personal attitude with cognitive and affective states [33]. The third definition is more comprehensive as it involves the relationships between the environment and personal environmental satisfaction [35]. Previous studies demonstrated that personal traits, individual characteristics, and individuals’ socioeconomic status were associated with satisfaction [36,37,38]. 

Therefore, studying the relationship between the individual co-production of the community environment and personal satisfaction with the community environment among elderly adults in urban is important to improve the quality of environmental governance. This study uses cross-section survey data from elderly residents in Shanghai, China. Our study aimed to examine the relationship between co-production levels and satisfaction with the community environment among older residents.

## 2. Materials and Methods 

### 2.1. Research Site

This survey was conducted in Shanghai, China, a city with the highest level of aging in China. The urban aging problem in downtown Shanghai is even more severe (39.68% of residents in Chongming District were aged 60 or above, Hongkou of 33.21%, Yangpu District 31.78%, Jingan District 31.57%, Putuo District 30.56%, Changning District 29.10%, Xuhui District 28.71%, Huangpu District 26.60%, and Jinshan District 23.57%). Since 2009, Shanghai has built a series of age-friendly communities to cope with the wave of aging, and there were 35 national models of age-friendly communities.

**Figure 1:**
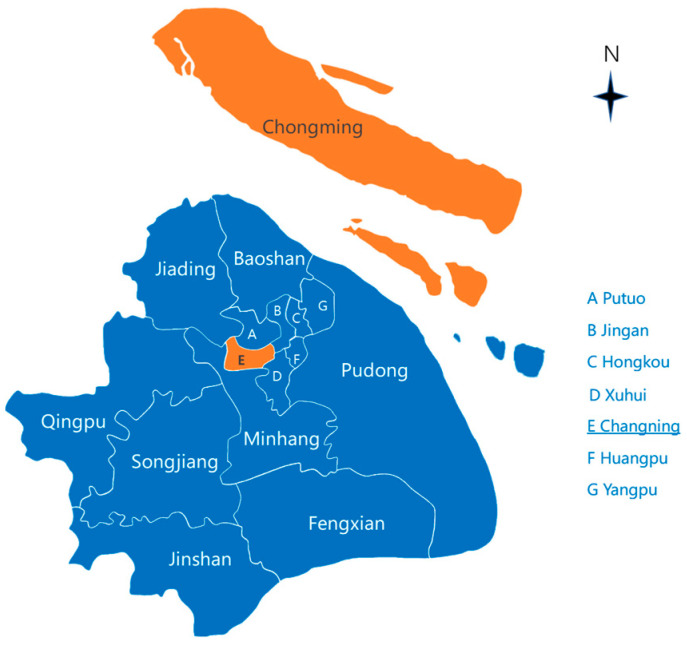
The locations of two selected districts in Shanghai.

As a part of the Healthy Shanghai Initiative, Chongming District (a suburban district) and Changning District (a core district) were selected for this survey, which is shown in the simple geographical map (Figure 1). Specifically, 2 of the best models of age-friendly communities in Chongming District (Changzheng community and Yuejin community) and Changning District (Lvba community and Songer community) were surveyed. 

### 2.2. Study Design and Participants

The cross-sectional survey was conducted from 8–15 November 2022 using the snowball sampling method. Survey notices were posted on the bulletin boards of the sample communities, and the residents and community workers were encouraged to spread the word and forward it to others. Questionnaires were distributed to elderly residents personally by trained researchers and collected after completion on the same day. The survey was completed on a voluntary and anonymous basis. All participants involved in this survey provided informed consent before they started answering questions.

The eligibility criteria for the survey participants included: (1) aged 60 or above; (2) Chinese ethnicity; (3) lived in the community for more than one year; (4) being able to read and understand the contents of the questionnaire and could answer the questionnaire independently. All questions were mandatory to answer to avoid missing items in the questionnaire. This study protocol was reviewed and approved by the Shanghai Jiao Tong University Ethics Committee (approval number: H2022106P).

The sample size was calculated using the formula: SS = Z^2^ × (P) × (1 − P)/C^2^, in which SS is the sample size, Z is the abscissa of the normal curve that cuts off an area α at the tails, C is the desired level of precision, P is the estimated proportion of an attribute that is present in the population [39]. The value for Z is found in statistical tables, which contain the area under the normal curve. In this survey, we assumed P = 0.5, desired α as 0.05, and C = 5%. It was calculated that SS = 385. If 10% of those invited would refuse to participate in this study, at least 428 (=385/0.9) participants had to be included. 

### 2.3. Measures

#### 2.3.1. Community Environment Co-Production and Environmental Satisfaction

A self-developed questionnaire was used to measure levels of community environment co-production and environmental satisfaction. In this study, we focused on environmental satisfaction in the community and neighborhood environments, which the residential co-production could modify. We used a modified Delphi method to develop the questionnaire [40,41,42]. Recommendations for optional items were extracted from the literature (PubMed and Web of Science database) and were translated into potential items pool to be screened by a multidisciplinary expert panel.

The rating procedure took place between 5 August and 20 September 2022. Our expert panel consisted of 13 experts in China with environmental governance experience (6 public health experts, 4 social work experts, and 3 healthcare policy experts; 7 experts had doctor’s degrees, and 3 experts had master’s degrees). The average authoritative coefficient was 0.88 ± 0.07. 

After two rounds of Delphi consultation, 18 items for environmental satisfaction (13 sub-indices and 5 overall indices, including dimensions of physical community environment, community information service, social environment, emotion support, and policy implementation at the community level), and 2 items for co-production (I always take part in coproducing of community environment governance; every resident should participate the co-product process), were identified for elderly urban residents. A Likert scale ranging from 1 (not at all/very dissatisfied/strongly disagree) to 5 (very often/very satisfied/strongly agree) was added for each item. The total score was 90 for satisfaction and 10 for co-production, respectively. Co-production and environmental satisfaction scores were treated as independent and dependent variables. The Cronbach’s α of the environmental satisfaction questionnaire in our samples was 0.97 and 0.86 for the co-production questionnaire, respectively.

#### 2.3.2. Covariates

We controlled a set of individual characteristics that might play a significant role in moderating environment satisfaction, including general health status, affective commitment to the community, and essential socio-demographic characteristics.

The general health status was evaluated through the EuroQol-Visual Analogue Scale (EQ-VAS), a 20-cm thermometer-like scale [43]. The self-reported scale measures general health status from endpoints of 0 (worst imaginable health status) to 100 (best imaginable health status).

Affective commitment to the community was evaluated through a question: “Do you agree you are a part of the community”? [44] A five-point Likert scale measures responses to this question, ranging from 1 (strongly disagree) to 5 (strongly agree).

Physical activity was measured through the Physical Activity Rating Scale-3 (PARS-3), a three-item self-reported scale comprising physical activity intensity, duration, and frequency [45]. Each item is rated from 1 to 5, and the total score (i.e., exercise volume), ranging from 0 to 100, is computed by the equation: intensity × (duration-1) × frequency. When the total score is less than 20, it will be defined as light, and from 20 to 42 will be described as a medium, while more than 42 will be defined as a high exercise. 

Essential individual socio-demographic characteristics of the elderly urban residents were collected in this study, including gender, age, educational background, marital status, party membership, average monthly household income, BMI (calculated based on self-report height and weight), sleep hours per night, and community name. This adjustment is feasible and aligned with previous studies [10,46]. 

### 2.4. Data Analysis

We used the one-sample K-S test to determine the normality of survey data. Descriptive analyses for community environment co-production, the satisfaction of the environment, the participants’ characteristics, and other related factors were conducted. The univariate analyses of factors associated with satisfaction were conducted with a *t*-test, analysis of variance (ANOVA), Pearson correlation, or Spearman correlation analysis, as appropriate.

In this study, as the participants were nested in four communities, we treated the individual characteristics as level 1 and communities as level 2. The associations between co-production and satisfaction with the environment were examined through the multilevel regression if the Intraclass Correlation Coefficient (ICC) is more than 0.1 in the null model [47]. In model 1, we only involved the co-production scores in level 1, while in model 2, we involved co-production scores and all other significant related factors which were determined in the univariate analyses.

Data analyses were carried out using STATA software version 16.0 (Stata Corporation, College Station, TX, USA). We set the significance level at the *p*-value of 0.05 (2-tailed).

## 3. Results

In total, 485 elderly urban residents completed the survey, and 480 valid questionnaires without logical errors were included in the statistical analysis. 

The mean age of the participants was 69.83 ± 6.11 years, and 58.13% of them were female. Two-fifths (41.25%) had a junior middle school educational background, most (80.0%) were married, and 58.75% lived with their spouse. The average monthly household income was 5000–9999 RMB (712–1425 US dollars; 52.08%). The satisfaction scores for the community’s environment were 76.82 ± 12.40 out of a total score of 90, or 85.35% satisfaction degree. At the same time, the median of the co-production, affective commitment, and EQ-VAS was 8, 5, and 87.5, respectively. Table 1 shows detailed information. 

In univariate analysis, educational background (F = 2.94, *p* = 0.033), living situation (F = 4.23, *p* = 0.006), different levels of physical activity (F = 4.25, *p* = 0.015), and in various communities were significantly associated with environmental satisfaction (see Table 2).

In correlation analysis, sleep hours per night (*r* = 0.15), co-production scores(*r* = 0.86), affective commitment(*r* = 0.80), and EQ-VAS (*r* = 0.27) were significantly associated with environmental satisfaction (all *p* < 0.001). As general health status was an essential output of the community environment, we further analyzed the relationship between EQ-VAS and other factors. As shown in Table 3, EQ-VAS was negatively related to age (*r* = −0.18) and positively associated with sleep hours (*r* = 0.23), co-production (*r* = 0.24), and affective commitment (*r* = 0.19), and especially positively associated with environmental satisfaction (*r* = 0.27), and all *p* < 0.001. The variance inflation factors (VIF) of these variables were all less than 3, indicating low collinearity among them [48].

In the multilevel linear regression, the ICC in the null model was 0.264, demonstrating that multilevel linear regression was necessary for further analysis. In model 1, co-production was significantly associated with environmental satisfaction (β = 6.71, *p* < 0.001), and the ICC decreased to 0.054. In model 2, only co-production (β = 4.68, *p* < 0.001), affective commitment (β = 6.17, *p* < 0.001), and EQ-VAS (β = 0.06, *p* = 0.002) were significantly associated with environmental satisfaction, while the ICC further decreased to 0.003. In the educational background, college or above was negatively associated with environmental satisfaction but was insignificant (*p* = 0.052). Table 4 shows detailed information.

## 4. Discussion

To our best knowledge, this survey was one of the first to study the relationship between community environment co-production and community environmental satisfaction among urban elderly residents in China. Multilevel regression analyses showed that co-production, affective commitment to the community, and personal general health status were significantly associated with environmental satisfaction. This study demonstrated that co-production was an independent factor contributing to environmental satisfaction. 

Implementing and measuring co-production in public service activity is challenging since it may occur asymmetrically [49]. Some scholars regard co-production in a wide range of activities with any degree or form in public service [46]. Citizens can contribute their inputs such as resources, skills, interactive communication, and necessary negotiation. As a conceptual measure, it is a consensus that co-production is a widespread public service delivery phenomenon, as it can be defined as simple participation, a form of public involvement, and individual investors. As this survey showed, the elderly residents in Shanghai had a very high degree of co-production in community environment governance.

This survey found that resident co-production was positively associated with environmental satisfaction, which was aligned with other studies. In healthcare settings, co-production was considered a critical driver for client satisfaction in German daycare centers [50]. Taylor-Phillips et al. (2014) revealed that co-production was associated with higher decision satisfaction among staff employed at National Health Service (NHS) [51]. In education, Cicatiello et al. (2021) used European cross-country microdata to reveal that parents’ co-production in homeschooling strongly correlated with their satisfaction with online schooling during the COVID-19 pandemic [52]. Affective commitment or consumer motivation might mediate the relationship between co-production and satisfaction [53]. Meanwhile, as few studies have focused on this relationship’s mechanism, further research is needed to uncover the potential mechanism.

We found that residents had a high degree (85.35%) of environmental satisfaction overall, which was moderately higher than other studies in China. For example, Wang et al. (2019) revealed that environmental satisfaction was 3.8 (76%) out of a total score of 5 among adult urban residents in Guangzhou, China [28]. Shen et al. (2019) found that 57.1% of adult citizens in Shanghai suburbs were satisfied or very satisfied with their community’s environment [54]. This gap may be due to the difference in research sites. In this study, we selected the top age-friendly communities with likely better services and satisfaction. Another reason is the difference in measurement tools and sampling population.

One finding from this study needs to be highlighted, which is that environmental satisfaction was positively associated with an individual’s general health status (EQ-VAS). Residents with a more positive environmental satisfaction showed healthier or were more optimistic. Moreover, residents with lower environmental satisfaction reported either lower optimism or less social support. Meanwhile, it is necessary to keep in mind that optimism is a dispositional characteristic that can influence an individual’s evaluation of the community’s environment. This result is in line with Ruiz et al. (2019) [55], showing the importance of environmental satisfaction for health. 

Although we explored the relationship between community environment co-production and environmental satisfaction, and our findings may improve the development of an age-friendly community, several limitations to the study also need to be mentioned. First, this cross-sectional survey could not address the causal relationship between co-production and environmental satisfaction. To date, there is limited information on environmental co-production and environmental satisfaction in age-friendly initiatives, so further research relating to this scope, and barriers in this context, is urgently needed. Second, this study did not control factors such as *Hukou* (household registry), homeownership, and duration of residence, which may affect environmental satisfaction. Third, all elderly urban residents were recruited from Shanghai, China. The selected cases might not be representative of the whole city. As a result, the generalizability of the research conclusions is limited. Fourth, this study cannot rule out recall and response bias. Finally, because of the eligibility criteria, some potential participants, especially those with more advanced age or with cognitive impairments, may not be able to participate. This might also limit the generalizability of our findings. 

## 5. Conclusions

In this study, co-production was significantly associated with community environment satisfaction, affective commitment, and individual health status were also significant factors associated with environment satisfaction. 

The findings suggest that resident co-production is important in community environment governance and should be encouraged when developing age-friendly communities for the elderly. The findings may also guide further research to develop a theoretical framework for measuring the value of co-production. Meanwhile, as related factors of co-production are the policy landscape, future studies should explore the influences on residents’ co-production and its implications.

Local government actions involving residents’ co-production to build age-friendly communities will be important. Hence, we suggest that policymakers be aware of these massive benefits of co-production and include residents in service processes. Across different stages of public service, including planning, policy-making, production, delivery, monitoring, and improvement, local governments should create more opportunities for the co-production of community residents.

Governments alone cannot provide all public services. Therefore, it is necessary to receive the assistance of the public to improve public service quality. In this process, both human and financial resources are determinants of co-production. The performance of the local governments is the determinant of residents’ co-production, while the economic resources provided by local governments are also essential for co-production. Local governments should pay more attention to human and financial resources to ensure the residents’ co-production. Future studies should uncover how local governments stimulate residents’ co-production, why co-production fails or succeeds, and the relationship between impacts and drivers of co-production.

Community environment planning, which can involve opportunities for residents’ co-production, has a crucial role in improving communities forward to address public health challenges and create livable communities for the elderly. A comprehensive way must link the community environment with the resident’s health and reorganize the resident’s co-production (public engagement and opportunity). The key is to keep a balance between residents’ co-production, public services, and the community environment.

## Figures and Tables

**Table 1:** Basic characteristics of 480 participants.

Characteristic		N	%
Gender			
	Male	210	41.88
	Female	279	58.13
Education			
	Primary school or below	58	12.08
	Junior middle school	198	41.25
	High school	147	30.63
	College or above	77	16.04
Marital status			
	Not married	14	2.92
	Married	384	80.0
	Divorced	22	4.58
	Widowed	60	12.50
Living situation			
	Alone	86	17.92
	With spouse	282	58.75
	With children	54	11.25
	With spouse and offspring	58	12.08
Average monthly household income			
	Low (<3000 RMB)	33	6.88
	Middle (3000–4999 RMB)	98	20.42
	Upper middle (5000–9999 RMB)	250	52.08
	High (10,000–19,999 RMB)	89	18.54
	Very high (20,000- RMB)	10	2.08
Physical activity level			
	Low	364	75.83
	Medium	106	22.08
	High	10	2.08
Community			
	Lvba	151	31.46
	Changzheng	89	18.54
	Yuejin	120	25.0
	Songer	120	25.0
		Mean	SD
Age (years)		69.83	6.11
BMI		23.55	3.27
Sleep hours		6.56	1.25
Environment satisfaction		76.82	12.40
		Median	IQR
Coproduction		8	2
Affective commitment		5	1
EQ-VAS		87.5	15

**Table 2:** Univariate analysis of factors associated with environmental satisfaction.

Variable		Environmental Satisfaction Score		
		Mean (SD)	F/t	*p*
Gender			−0.22	0.822 ^a^
	Male	76.66 (12.81)		
	Female	76.92 (12.12)		
Education			2.94	0.033 ^b^
	Primary school or below	79.19 (12.45)		
	Junior middle school	77.99 (11.93)		
	High school	74.61 (12.71)		
	College or above	76.22 (12.49)		
Marital status			0.84	0.472 ^b^
	Not married	78.21 (13.66)		
	Married	76.42 (12.43)		
	Divorced	80.09 (11.94)		
	Widowed	77.85 (12.12)		
Living situation			4.23	0.006 ^b^
	Alone	81.06 (10.63)		
	With spouse	75.91 (12.53)		
	Whit offspring	75.33 (13.83)		
	Whit spouse and offspring	76.31 (11.80)		
Average monthly household income			0.81	0.521 ^b^
	Low (<3000 RMB)	80.21 (12.05)		
	Middle (3000–4999 RMB)	76.68 (12.30)		
	Upper middle (5000–9999 RMB)	76.81 (12.55)		
	High (10,000–19,999 RMB)	75.69 (12.31)		
	Very high (20,000- RMB)	77.20 (12.02)		
Physical activity level			4.25	0.015 ^b^
	Light	75.95 (12.37)		
	Medium	79.90 (11.62)		
	High	75.70 (17.18)		
Community			57.83	<0.001 ^b^
	Lvba	76.25 (10.73)		
	Changzheng	79.90 (9.93)		
	Yuejin	84.84 (10.24)		
	Songer	67.22 (11.43)		

^a^ *t*-test; ^b^ ANOVA.

**Table 3:** Correlation analysis for environmental satisfaction and EQ-VAS.

Variable	Environmental Satisfaction	EQ-VAS
Age (years)	−0.07 ^a^	−0.18 ^b^
BMI	0.05 ^a^	−0.01 ^b^
Sleep hours	0.15 ^a^	0.23 ^b^
Co-production	0.86 ^b^	0.24 ^b^
Affective commitment	0.80 ^b^	0.19 ^b^
Environmental satisfaction	1.0 ^a^	0.27 ^b^
EQ-VAS	0.27 ^b^	1.0 ^b^

^a^ Pearson correlation; ^b^ Spearman correlation.

**Table 4:** Association of co-production and environmental satisfaction.

		Null Model			Model 1			Model 2		
Variable		*β*	95% CI	*p*	*β*	95% CI	*p*	*β*	95% CI	*p*
Level 1	Co-production				6.71	[6.28,7.14]	<0.001	4.68	[4.09,5.28]	<0.001
	Education									
	Primary school or below							Reference		
	Junior middle school							0.39	[−1.33,2.12]	0.654
	High school							−0.51	[−2.33,1.32]	0.588
	College or above							−2.02	[−4.07,0.02]	0.052
	Living situation									
	Alone							Reference		
	With spouse							−0.49	[−1.94,0.95]	0.502
	With offspring							−1.36	[−3.36,0.63]	0.181
	With spouse and offspring							−0.69	[−2.67,1.30]	0.498
	Physical activity level									
	Light							Reference		
	Medium							−0.87	[−2.18,0.43]	0.189
	High							1.14	[−2.56,4.85]	0.545
	Sleep hours							−0.22	[−0.65,0.22]	0.333
	Affective commitment							6.17	[4.94,7.41]	<0.001
	EQ-VAS							0.06	[0.02,0.10]	0.002
Level 2	Community	40.60	[9.83,167.74]	<0.001	2.30	[0.44,11.99]	<0.001	0.11	[0.00,193.70]	0.383
	Log-likelihood	−1824.1435			−1569.6458			−1522.3663		
	ICC	0.264			0.054			0.003		

## Data Availability

Data are available upon reasonable request.
